# Comparison of online and in-person cognitive behavioral therapy in individuals diagnosed with major depressive disorder: a non-randomized controlled trial

**DOI:** 10.3389/fpsyt.2023.1113956

**Published:** 2023-04-28

**Authors:** Nazanin Alavi, Elnaz Moghimi, Callum Stephenson, Gilmar Gutierrez, Jasleen Jagayat, Anchan Kumar, Yijia Shao, Shadé Miller, Caitlin S. Yee, Anthi Stefatos, Maedeh Gholamzadehmir, Zara Abbaspour, Amirhossein Shirazi, Tessa Gizzarelli, Ferwa Khan, Charmy Patel, Archana Patel, Megan Yang, Mohsen Omrani

**Affiliations:** ^1^Department of Psychiatry, Queen’s University, Kingston, ON, Canada; ^2^Centre for Neuroscience Studies, Queen’s University, Kingston, ON, Canada; ^3^OPTT Inc., Toronto, ON, Canada; ^4^Department of Psychology, University of Toronto, Toronto, ON, Canada

**Keywords:** mental health, depression, psychotherapy, cognitive behavioral therapy, online mental health, internet therapy, electronic, mental health care

## Abstract

**Objective:**

The increased prevalence of major depressive disorder (MDD) amid the COVID-19 pandemic has resulted in substantial growth in online mental health care delivery. Compared to its in-person counterpart, online cognitive behavioral therapy (e-CBT) is a time-flexible and cost-effective method of improving MDD symptoms. However, how its efficacy compares to in-person CBT is yet to be explored. Therefore, the current study compared the efficacy of a therapist-supported, electronically delivered e-CBT program to in-person therapy in individuals diagnosed with MDD.

**Methods:**

Participants (*n* = 108) diagnosed with MDD selected either a 12 week in-person CBT or an asynchronous therapist-supported e-CBT program. E-CBT participants (*n* = 55) completed weekly interactive online modules delivered through a secure cloud-based online platform (Online Psychotherapy Tool; OPTT). These modules were followed by homework in which participants received personalized feedback from a trained therapist. Participants in the in-person CBT group (*n* = 53) discussed sessions and homework with their therapists during one-hour weekly meetings. Program efficacy was evaluated using clinically validated symptomatology and quality of life questionnaires.

**Results:**

Both treatments yielded significant improvements in depressive symptoms and quality of life from baseline to post-treatment. Participants who opted for in-person therapy presented significantly higher baseline symptomatology scores than the e-CBT group. However, both treatments demonstrated comparable significant improvements in depressive symptoms and quality of life from baseline to post-treatment. e-CBT seems to afford higher participant compliance as dropouts in the e-CBT group completed more sessions on average than those in the in-person CBT group.

**Conclusion:**

The findings support e-CBT with therapist guidance as a suitable option to treat MDD. Future studies should investigate how treatment accessibility is related to program completion rates in the e-CBT vs. in-person group.

**Clinical Trial Registration:**

ClinicalTrials.Gov Protocol Registration and Results System (NCT04478058); clinicaltrials.gov/ct2/show/NCT04478058.

## 1. Introduction

Major depressive disorder (MDD) is one of the most pervasive and debilitating mental health conditions and has a lifetime prevalence of 8% (1). The disorder is characterized by impairments in mood, affect, and motivation and is associated with cognitive dysfunction, reduced quality of life, and low psychosocial functioning (2, 3). The COVID-19 pandemic resulted in a global mental health crisis and increased rates of mental health disorders, including MDD (4). Indeed, the prevalence of depressive symptoms has increased (14.6–48.3%) when compared to one year before the pandemic (3.6–7.2%) (5). Despite these trends, the current healthcare system has insufficient capacity to accommodate the rapidly rising mental health care demands (6). Prior to the pandemic, the majority of mental health treatments were in-person—a delivery method that is often inaccessible, inefficient, and costly (7). To mitigate accessibility issues faced during the pandemic, use of online mental health care increased (8). Many traditionally in-person psychotherapies have been adapted to a digital format and are administered through telephone, internet, or mobile applications. This pivot enables broader access to scalable, affordable, and evidence-based therapies (9).

These adaptations have also extended to therapies like cognitive behavioral therapy (CBT). As the gold standard treatment for MDD in Canada (10), CBT mitigates symptoms by focusing on behavioral activation and cognitive restructuring (11). Specifically, individuals are taught strategies and skills to enhance awareness of their interconnected thoughts, emotions, and behaviors. Although this therapy is highly effective for managing depression (12), in-person delivery can be resource and time-intensive (13). A round of therapy usually consists of 12–15 one-hour sessions with a therapist, which can substantially increase waitlist times and treatment costs (14). Language or cultural barriers, exposure to public stigma, privacy concerns, and inflexible time schedules can also deter individuals from seeking care (13). Conversely, electronically delivered CBT (e-CBT) can address many of these barriers (15) while offering results comparable to in-person treatment (16–19).

Although digital mental health interventions have existed long before the pandemic, this unforeseen moment in time became a suitable case study on how to use digital health (20). These tools have great potential in improving the accessibility, availability, and scalability of evidence-based mental health care in a variety of populations and mental health concerns (21–26). Although online psychotherapy has been successful in mitigating psychosocial problems during the COVID-19 pandemic (27), its contribution to equitable health care has generated enthusiasm within the research and commercial community (28, 29). However, a robust shift towards the provision of online mental health treatment requires a good understanding of the treatments and how individuals interact with them (30). Currently, despite the acceptability and effectiveness of these treatments, high heterogeneity has been observed in the current body of studies (31).

Over the last few years, different approaches to e-CBT delivery have been investigated, including self-help (self-directed, no therapist), guided self-help (clinician providing limited support), and fully supervised (clinician providing weekly support) methods (32). The literature indicates that methods with greater therapist contact are typically more effective than those without (33). Although the potential for scalability is substantially greater in online than in-person interventions (34), online care with limited therapist contact may adversely impact clinical outcomes. Therefore, the current study compared the relative effectiveness of the novel therapist-guided e-CBT program and gold-standard in-person CBT on MDD symptomatology. The study also assessed patient compliance in the two treatment groups. The structured e-CBT program includes weekly text-based therapist guidance and is delivered via a secure platform through the Online Psychotherapy Tool (OPTT) (28, 35, 36).

## 2. Methods

### 2.1. Study design

A study protocol was previously published that provides a comprehensive overview of the research methods used in this study (36). Briefly, this non-randomized controlled trial study contained an e-CBT treatment arm and an in-person CBT control arm. Participants were provided with a verbal description of each treatment type by a research assistant on the team and given the option to select which to receive (e-CBT or in-person CBT). This process was meant to simulate a real-world setting where patients have autonomy over their care delivery format. Program efficacy was assessed using depressive symptomatology and quality of life questionnaires at pre-, mid-, and post-intervention. The study was reviewed by Queen’s University Health Sciences and Affiliated Teaching Hospitals Research Ethics Board for ethical compliance (File #: 6020045). This trial was registered through ClinicalTrials.Gov Protocol Registration System (NCT04478058).^1^

### 2.2. Sample size and recruitment

The sample size was calculated based on the effect sizes of previous online psychotherapy studies (Hedges’ *G* = 0.86) (35, 36). Given this effect size, a power of 0.8, a significance level of *p* = 0.05, a 45% completion rate from our previous studies (35, 36) and a lower-end estimate of around 30% disorder prevalence is accounted for, 23 study completers, translating into approximately 50 participants in each arm, would be enough to detect significant effects.

Recruitment occurred in outpatient psychiatry clinics at Hotel Dieu Hospital, Kingston General Hospital, and Providence Care Hospital, all located in Kingston, Ontario, Canada and through self, family doctors, specialists, and clinician referrals. Screening was conducted by a research assistant on the team and verbal consent was provided by all recruited participants (*n* = 113) before study commencement. Initial assessments were conducted by a psychiatrist on the research team either in-person or through video conference. Participants were evaluated for eligibility criteria and to confirm a diagnosis of MDD based on the Diagnostic and Statistical Manual of Mental Disorders, 5th Edition (DSM-5) criteria (37).

### 2.3. Study eligibility

Participants were recruited if they were 18 years or older at the start of the study, had an MDD diagnosis according to DSM-5 guidelines, had the ability to consent, and could speak and read English. Participants in the e-CBT arm were also required to have consistent and reliable access to the internet. Those in the in-person CBT arm had to have access to transportation to and from the hospital where the CBT sessions were conducted.

Participants were excluded from the study if they explicitly indicated during screening that they experienced symptoms of active psychosis, acute mania, severe alcohol or substance use disorder, and/or active suicidal or homicidal ideation. Additionally, prospective participants should not have received any form of psychotherapy within the past 12 months to avoid confounding treatment efficacy effects (38). Pharmacotherapy was not an excluding criterion if the participant continued with the same drug and dosage six weeks before and for the entirety of the study. Following the initial assessment, eligible participants were presented with both treatment options (e-CBT or in-person CBT) and were asked to select their treatment preference.

### 2.4. Interventions

#### 2.4.1. Cognitive behavioral therapy structure

Both CBT formats provided participants with skills to develop coping strategies and effective thinking patterns. The in-person and e-CBT content were intended to mirror one another and focused on: (1) an introduction to depression and the 5-part model (39), which encourage conceptualization of a specific situation or environment through an interlink of thoughts, feelings, physical reactions, and behaviours, (2) the Thought Record, which challenges negative thoughts by teaching individuals to observe the evidence supporting a thought and considering an alternative or balanced thought, and (3) behavioral strategies to help individuals become more engaged in their day-to-day activities and to cope with stressful situations effectively. Strategies included breathing techniques, activity scheduling, planning behavioral experiments, creating action plans, and developing distraction techniques.

#### 2.4.2. Online cognitive behavioral therapy

The depression module was designed based on the content that was clinically validated in a previous trial using email as the primary form of communication and therapy delivery (35). Each of the 12 weekly fixed-structured sessions consisted of approximately 30 interactive slides. The therapy was delivered through a secure, online psychotherapy delivery platform, OPTT.^2^ Through the platform, therapists can schedule and assign pre-designed modules, structured homework assignments, and symptomatology questionnaires. Moreover, the platform possesses a chat feature where participants and therapists can communicate feedback, check-ups, questions, and concerns. Once participants received a session, they were provided with a deadline to submit the corresponding homework within the week. The homework was then reviewed by the therapist and personalized written feedback was submitted along with the following week’s session. Participants were also sent a maximum of three automated reminders to submit their weekly homework. If participants failed to submit their homework after the third reminder, they were contacted by the research assistant on the team and removed from the study once they confirmed they no longer wished to participate. To standardize the quality of care and develop scalable care delivery, therapists used pre-designed session-specific feedback templates to write their personalized feedback. These templates focused on validating patient time and effort, reviewing content covered in the homework submission, summarizing previous therapy session content, and discussing strengths and areas for improvement in the homework submission.

#### 2.4.3. In-person CBT

In-person CBT participants received 12 weekly one-hour sessions from a trained therapist and were assigned weekly homework assignments. The homework was delivered in a fillable paper format and was due at the start of their next session. Participants received verbal feedback from the therapist and discussed their previous week’s homework during the sessions. In-person treatment occurred at Hotel Dieu Hospital located in Kingston, ON, Canada.

### 2.5. Cognitive behavioral therapy therapists

All therapists were psychotherapy-trained research assistants with backgrounds and training in psychotherapy. The therapists were hired by the principal investigator (PI), a clinician–scientist with expertise in CBT and electronically delivered psychotherapy (23, 28, 35, 40–43). Therapists were also instructed to complete CBT courses and workshops as part of their training. The therapists completed feedback on practice homework, which was then reviewed by the PI. Therapists were supervised by the PI and other licensed psychotherapists on the research team to ensure adequate quality of work. All online written feedbacks were reviewed before submission. In the e-CBT group, pre-designed session-specific templates were used to write personalized feedback for patients. This strategy was meant to retain patient engagement while simultaneously reducing therapist time delivering care (44, 45). On average, therapists in this online program spend 15–20 min to complete each patient feedback (35). The feedback workflow for in-person sessions were also reviewed by the psychotherapists on the research team prior to the session to ensure the same standard of therapist care during in-person and online sessions.

### 2.6. Outcomes and data analysis

The primary outcomes measured were changes in depressive symptoms based on the Patient Health Questionnaire-9 (PHQ-9) and the Quick Inventory of Depressive Symptomatology Questionnaire-Self Report (QIDS-SR) (46, 47). The PHQ-9 and QIDS-SR are valid and reliable 9- and 16-items self-report questionnaires used to diagnose and assess the severity of depression (48, 49). Both instruments have good internal consistency and good convergent validity and display similar and acceptable psychometric properties when assessing symptom severity (49, 50). Quality of life changes was assessed using the Quality of Life and Enjoyment Questionnaire (Q-LES-Q) (51). The Q-LES-Q is a 14-item self-report measure with good test–retest liability (0.63–0.89) and internal consistency (0.90–0.96) that captures an individual’s satisfaction and enjoyment in different areas of daily functioning including physical health, subjective feelings, leisure time activities, social relationships, work, school/coursework, household duties, and general activities (51, 52). Scores are transformed onto a scale ranging from 0–100, with higher scores indicated greater perceived quality of life. Questionnaires were collected through OPTT (e-CBT group) and paper-based (in-person CBT group) at baseline (week 0), mid-treatment (week 6), and post-treatment (week 12). Secondary outcomes were the average number of sessions completed and dropout rates in the two treatment arms.

Initially, all data were examined for missing, nonsensical, and outlying variables that were more than 1.5 IQR below the first quartile or more than 1.5 IQR above the third quartile. Missing data were not imputed and were analyzed on a per-protocol basis. All analysis was performed at a two-tailed significance level of *α* = 0.05, except for when a Bonferroni correction was needed. Independent samples *T*-tests were performed to compare demographic information of program completers and dropouts and to identify possible differences. Baseline demographic and clinical characteristics of e-CBT and in-person CBT were also compared via chi-squared tests for categorical variables and independent sample *T*-tests for continuous variables. A 2 by 3 repeated measures analysis of variance (ANOVA) of the primary outcomes was also conducted to test for the effect of the treatment group (e-CBT or in-person CBT) on study outcomes over the course of the 12-week treatment (0, 6, and 12 weeks). An intention-to-treat analysis was used to evaluate the clinical effects of treatment on participants who withdrew prematurely. Linear mixed-effects models were also conducted with random effects as Patient ID and fixed effects as CBT Delivery Type and Time and their interaction. All statistical analyses were conducted using IBM SPSS Statistics for Mac, version 24 (IBM Corp., Armonk, NY, United States).

## 3. Results

### 3.1. Participants

**Figure 1:**
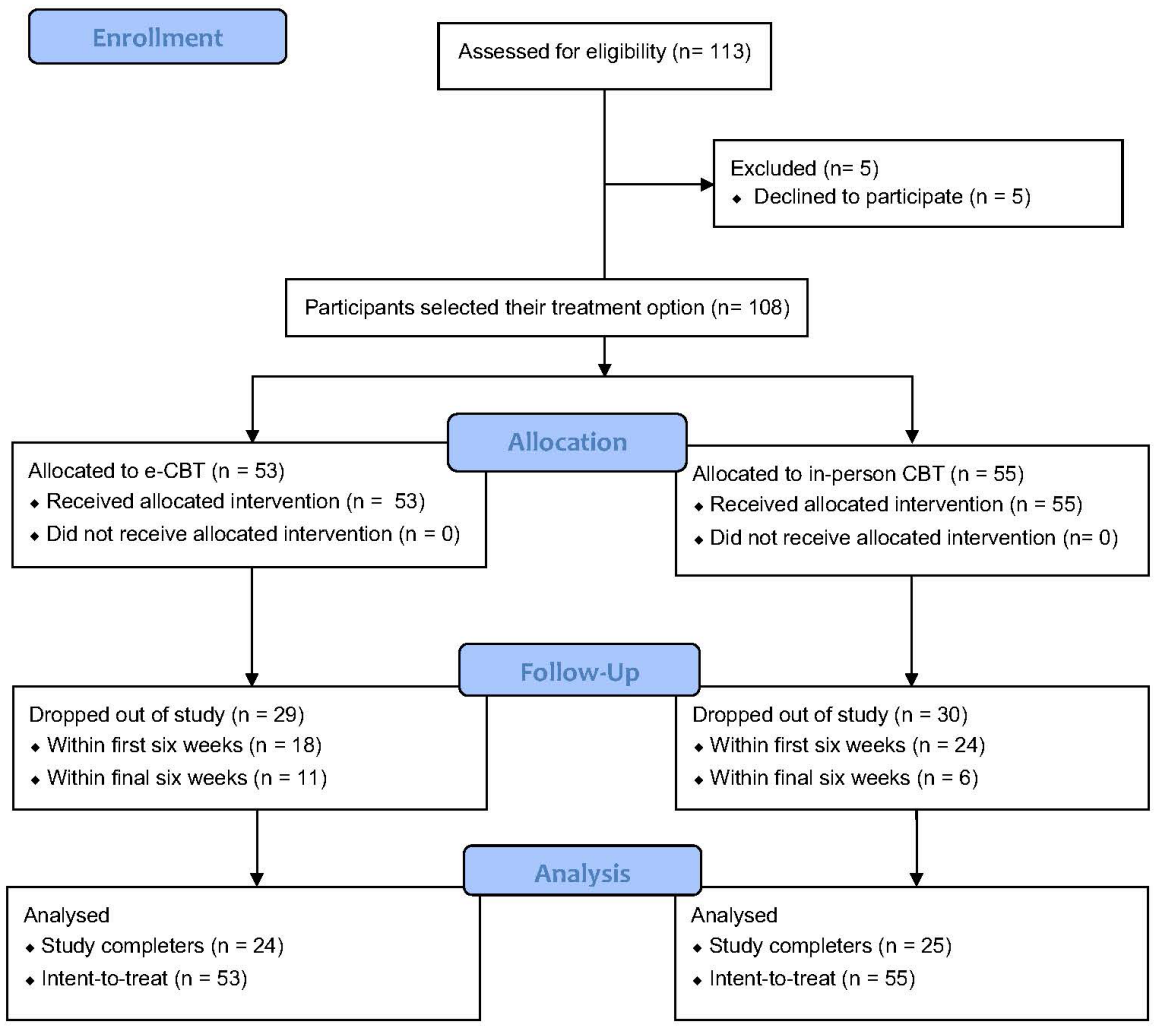
CONSORT flow diagram of study recruitment.

Of the recruited participants, 5 participants did not start the trial, resulting in a total sample size of *n* = 108 (e-CBT, *n* = 53; in-person CBT, *n* = 55). Participants were recruited from June 2019 to December 2021. Of the 53 participants recruited to the e-CBT group, 18 participants dropped out during the first six weeks, and 11 dropped out during the final six weeks, yielding 24 treatment completers who reached the study endpoint. From the in-person CBT participants (*n* = 55), 25 completed the study. Of those that dropped out, 24 participants were within the first six weeks of their sessions, and six participants were within the final six weeks of their sessions (Figure 1).

The total sample (*n* = 108) comprised mostly females (*n* = 70; 33 e-CBT; 37 in-person CBT). The average age of the e-CBT group was 38.6 (SD = 13.61) and in the in-person CBT group was 36.36 (SD = 11.61; Table 1). Student’s *T*-tests and Chi-square analyses did not indicate significant differences between the two groups on sex and age variables (Table 1).

**Table 1:** Demographics and characteristics of the sample, separated by treatment group, electronic cognitive behavioral therapy (eCBT) and in-person CBT (cognitive behavioral therapy).

	e-CBT (*n* = 53)	In-person CBT (*n* = 55)	Statistical analysis
Age (*n*, mean, SD)	48, 38.6, 13.61	55, 36.36, 11.61	*t*(93) = −0.850, *p* = 0.397
Sex (*n*, %)			*x*^2^ (2) = 5.466, *p* = 0.065
Female	33, 62.3%	37, 67.3%	
Male	15, 28.3%	18, 32.7%	
Did not indicate	5, 9.4%	0	
Program completers (*n*, %)	24, 45.3%	25, 45.5%	*x*^2^ (1) = 0.000, *p* = 0.986
Average number of sessions completed by dropouts (mean, SD)	3, 2.46	5, 2.48	*t*(57) = 2.625, *p* = 0.011

All *p* values are two-tailed and *α* = 0.05.

### 3.2. Clinical outcomes

#### 3.2.1. Baseline characteristics

Baseline clinical scores were significantly different between the two groups. Mean PHQ-9 scores in e-CBT participants were 16.08 (SE = 0.7) compared to 19.91 (SE = 0.73) in the in-person group (*p* < 0.001). Quality of life, as evaluated by the Q-LES-Q questionnaire, was significantly lower in the in-person group compared to the e-CBT group (Q-LES-Q mean, SE = 32.09, 1.32, and 36.31, 1.19 for in-person and e-CBT respectively, independent samples *T*-test *p* = 0.021). QIDS-SR scores were also significantly higher in the in-person group than in the e-CBT group (QIDS-SR mean, SE = 19.29, 0.63 and 14.61, 0.67 for in-person and e-CBT respectively, independent sample *T*-test *p* < 0.001) (see Supplementary material for descriptive statistics and analysis tables).

#### 3.2.2. Completer analysis

**Figure 2:**
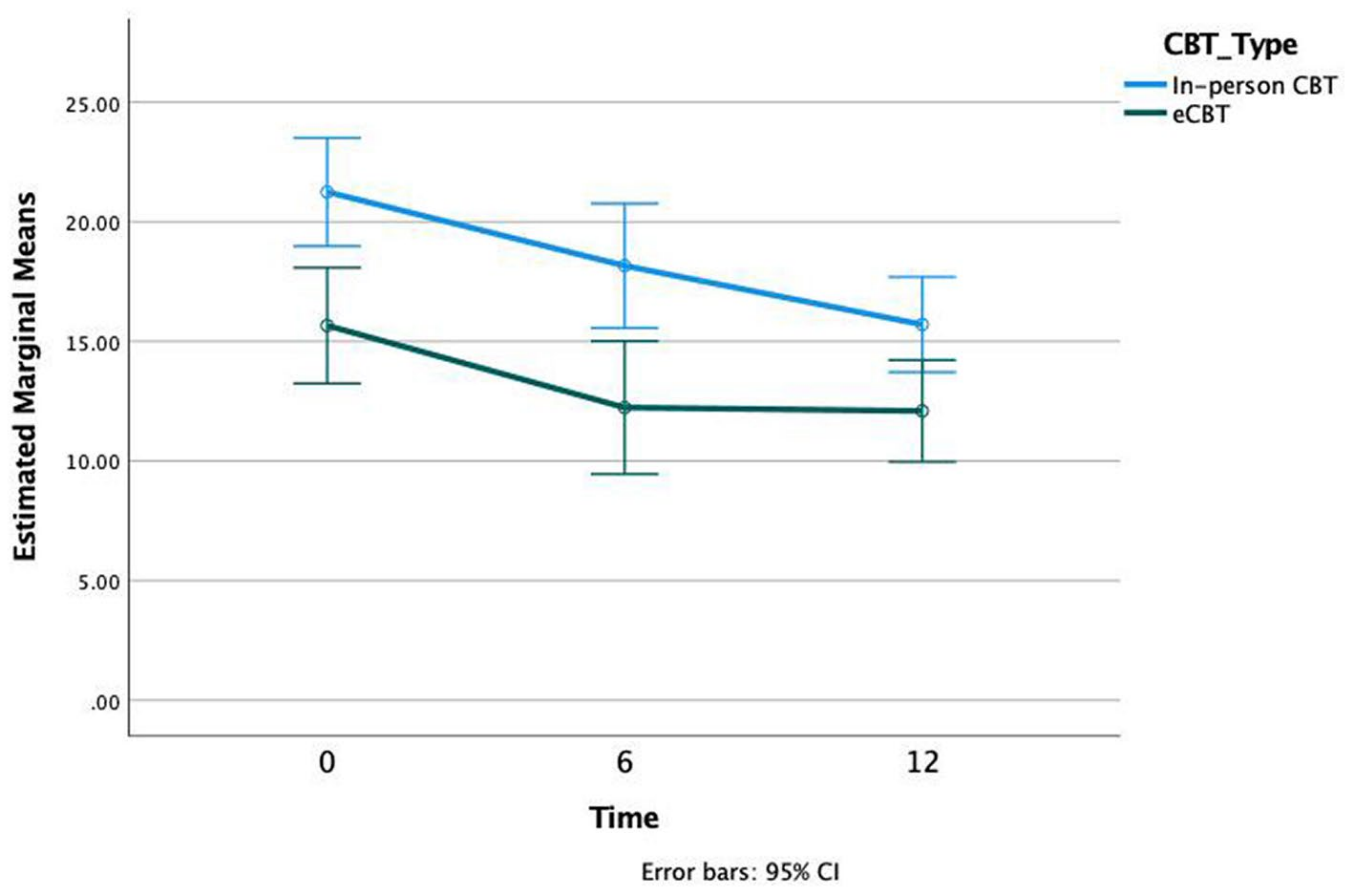
Patient Health Questionnaire (PHQ-9) scores at three time intervals of treatment, Weeks 0, 6, and 12, in both in-person and online cognitive behavioral therapy (e-CBT) treatment conditions.

CBT was significantly associated with lower PHQ-9 scores (*p* < 0.001, *df* = 2, *F* = 18.88). Despite the initial difference in PHQ-9 scores at the baseline across the two groups, the interaction of CBT effect across the two groups (i.e., effect size) was not significantly different across the two groups (*p* = 0.27, *df* = 2, *F* = 1.34). Additionally, *Bonferroni* post-hoc analysis demonstrated significant differences in PHQ-9 scores at 0 weeks vs. 6 weeks (*p* < 0.001) and 0 weeks vs. 12 weeks (*p* < 0.001), but not 6 weeks vs. 12 weeks (*p* = 0.260; Figure 2).

**Figure 3:**
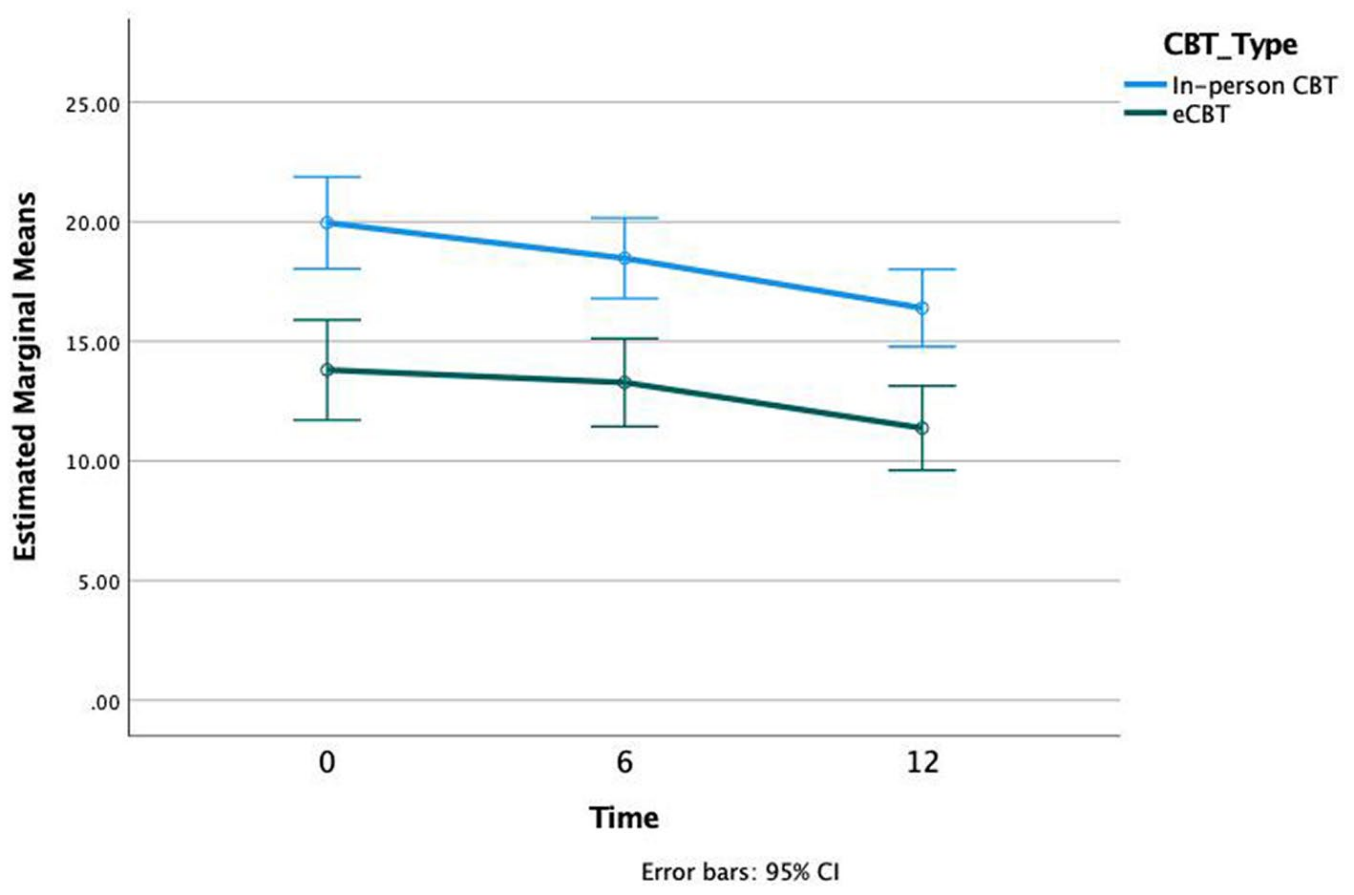
Quick Inventory of Depressive Symptomatology (QIDS-SR) scores at three time intervals of treatment, Weeks 0, 6, and 12, in both in-person and e-CBT treatment conditions.

With respect to QIDS-SR scores a significant association with CBT was observed (*p* < 0.001, *df* = 2, *F* = 18.46). The effect size was not significantly different across groups (*p* = 0.48, *df* = 2, *F* = 0.737). *Bonferroni* post-hoc analysis demonstrated significant differences in QIDS-SR scores at 0 weeks vs. 12 weeks (*p* < 0.001) and 6 weeks vs. 12 weeks (*p* < 0.001), but not 0 weeks vs. 6 weeks (*p* = 0.111; Figure 3).

**Figure 4:**
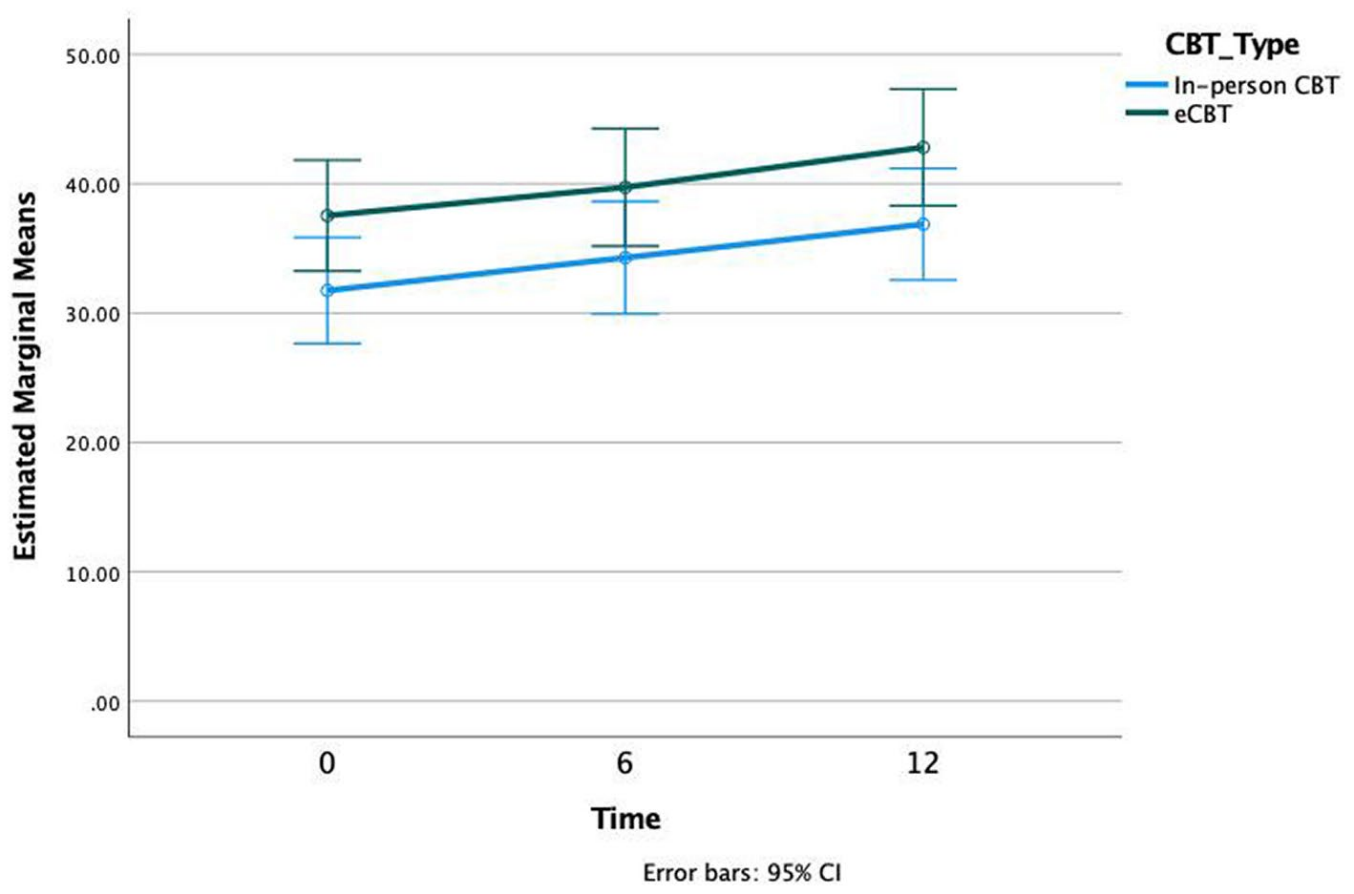
Quality of Life Enjoyment and Satisfaction (Q-LES-Q) scores at three time intervals of treatment, Weeks 0, 6, and 12, in both in-person and e-CBT treatment conditions.

Both treatment groups also demonstrated significant improvement in quality-of-life following CBT as evaluated by the Q-LES-Q questionnaire. CBT was associated with significantly increased Q-LES-Q scores (*p* < 0.001, *df* = 1.701, *F* = 14.01). The effect size was not significantly different across groups (*p* = 0.95, *df* = 2, *F* = 0.035). *Bonferroni* post-hoc analysis demonstrated significant differences in Q-LES-Q scores at 0 weeks vs. 6 weeks (*p* = 0.005), 6 weeks vs. 12 weeks (*p* = 0.047), and 0 weeks vs. 12 weeks (*p* < 0.001; Figure 4).

#### 3.2.3. Intent-to-treat analysis

An unstructured mixed-effect ANOVA analysis was used to evaluate intent-to-treat analysis (with CBT-type and evaluation times as fixed factors), which also included participants who did not complete the whole round of therapy. Similar to the previous analysis, a significant change in PHQ-9 score was observed across the groups (*p* < 0.001) and no significant difference between the effect size across the groups (*p* = 0.497) was observed. Unstructured mixed-effect ANOVA also revealed a similar improvement in quality of life as evaluated by the Q-LES-Q (*p* < 0.001), with no significant difference between the effect size across the groups (*p* = 0.96). There were also significant improvements in QIDS-SR scores (*p* < 0.001) and no significant difference between the effect size across the groups (*p* = 0.67; see Supplementary material for descriptive statistics and detailed analysis).

**Figure 5:**
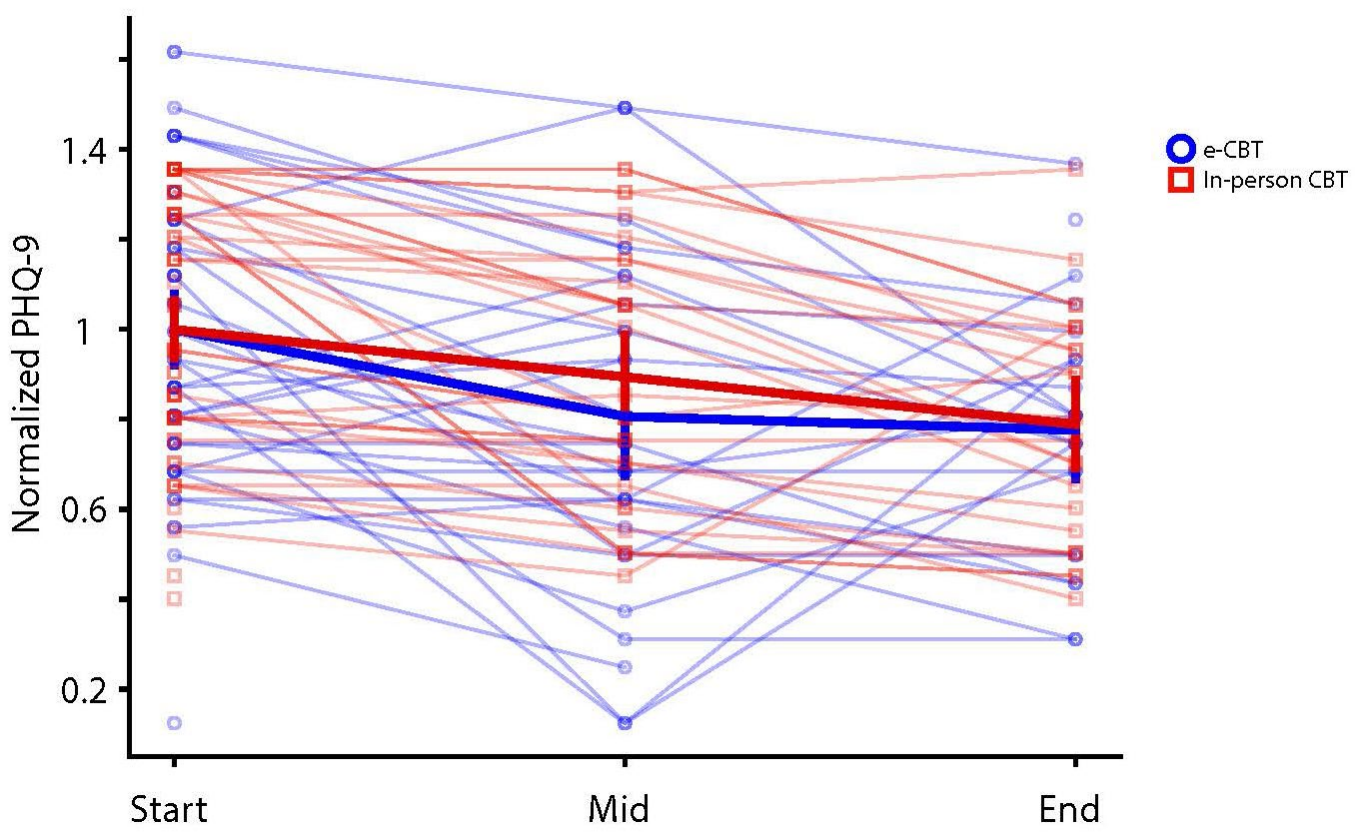
Individual and average (± 2SE) normalized PHQ-9 scores across e-CBT and in-person CBT groups over the three time intervals.

To ensure the effect sizes across the two groups were comparable despite baseline differences, the percent changes in PHQ-9 scores for each individual patients and across the two groups were analyzed. For this analysis, the initial PHQ-9 score of each patient was normalized to the average PHQ-9 score in their treatment group (Figure 5). By design, there is no significant difference between PHQ-9 scores in the beginning (average normalized PHQ-9 score in the in-person group = 1 and in the online group = 1, value of *p*(*t*-score) = 0.99 (<0.0001) for an independent sample *t*-test). The normalized PHQ-9 scores in each group were not significantly different in the mid or end point evaluations (average normalized mid- and end-PHQ-9 scores in the in-person group = 0.89 and 0.79 respectively, and in the online group = 0.81 and 0.78, value of *p*(*t*-score) = 0.31(1) and 0.87(0.16) respectively for independent sample *t*-tests).

#### 3.2.4. Patient compliance

**Figure 6:**
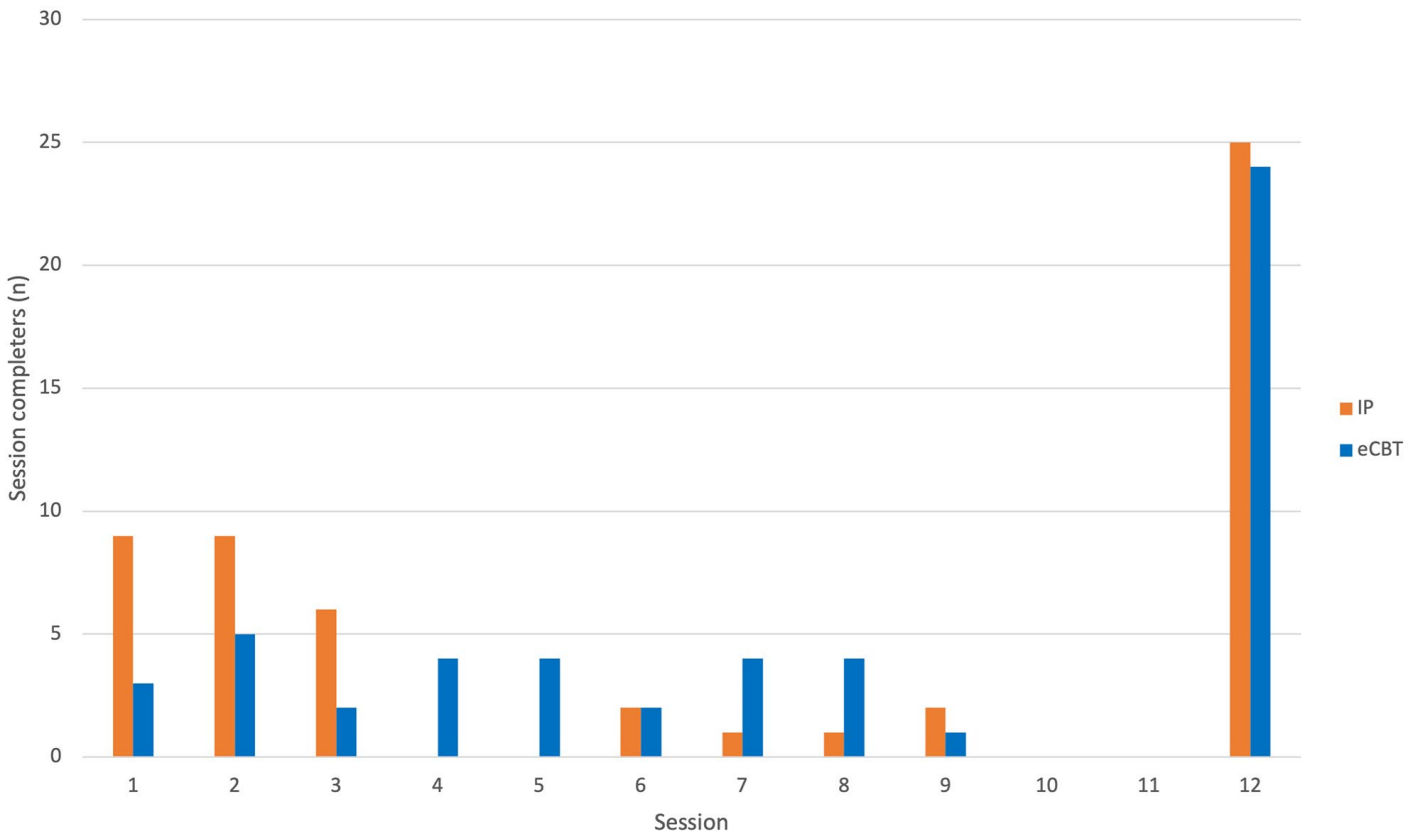
Frequency of participants’ last completed sessions organized by CBT delivery type.

While the number of patients completing the full round of therapy was comparable, there was a significant difference in patient compliance across the two groups. A Chi-square test indicated that the number of patients completing the full round of therapy (i.e., 12 sessions) was comparable across the two groups (45.45% in the in-person group and 45.28% of patients in the e-CBT group, *p* = 0.86). Independent samples *T*-test demonstrated that among the patients who dropped out of therapy, those in the e-CBT group completed significantly more sessions than those in the in-person group (*p* = 0.011; Table 1). On average, patients who dropped out of the study completed three sessions (SE = 0.44) in the in-person group and 4.65 sessions (SE = 0.46) in the e-CBT group. While patient dropout occurred uniformly across the first eight sessions in the e-CBT group, most patients in the in-person group dropped out within the first three sessions (Figure 6). Baseline PHQ-9 scores were not significantly different between treatment completers and non-completers (independent sample *t*-test, *p* = 0.480, *t* = −0.708). Further, no correlation was observed between PHQ-9 scores and the number of completed sessions (Pearson correlation, *r* = 0.058, *p* = 0.553).

## 4. Discussion

The current non-randomized controlled trial compared the effects of a 12 week therapist-guided e-CBT program to its in-person counterpart. Both e-CBT and in-person CBT groups yielded significant and comparable improvements in depressive symptom severity and quality of life. Reflecting real-life circumstances, the current study was one of the few to enable participants with MDD to select their treatment preferences. Although both CBT programs were efficacious, the study also highlights that patients with variable MDD severity may prefer different levels of therapist support.

In line with the current body of studies, the findings support the efficacy of therapist-guided CBT, independent of delivery type, in improving depressive symptoms and quality of life (53, 54). CBT primarily teaches patients skills and strategies to notice and restructure maladaptive thoughts and behaviours (55). Both programs successfully reduced clinical symptoms. While no correlation was observed between depression severity and compliance, participants in the e-CBT group, who had significantly lower depression severity, completed approximately 66% more sessions than those in the in-person group before dropping out. The convenience of e-CBT programs frequently described by patients (56–58) may also partly explain why participants in this group completed more therapy sessions. Moreover, the addition of therapist support to the online program may have contributed to attrition levels being on par with in-person therapy (59).

### 4.1. Limitations

An important limitation of the study was that it was non-randomized. This design made it challenging to discern conclusions on the superiority of one treatment type over the other. Furthermore, the apparent baseline differences indicate that any comparison between the two treatment types must be interpreted with caution. Since pervious randomized controlled trials have demonstrated relatively comparable efficacy of e-CBT to in-person therapy for depression (53, 60), it was important to determine how these trends may be similar or different under real-world circumstances. Providing participants with a choice gave the opportunity to assess treatment effectiveness. This strategy reflected a real-world model where participants had autonomy over their treatment delivery method. Baseline differences between participants in the treatment groups highlights the importance of patient-centered therapy and the need for future studies to account for symptom severity when allocating patients to the most appropriate treatment type.

In addition, although attrition rates were not significantly different between the groups, they stood at approximately 55%. Several factors may have contributed to these elevated dropout rates. The study was conducted during the COVID-19 pandemic, resulting in lockdowns and sporadic policy changes to social distancing laws. The inconvenience of navigating through these changes, particularly at a time of financial constraints, job insecurity, and childcare limitations, may have resulted in higher than usual dropout rates. Although participants in this study were not surveyed on the impact of COVID-19 on their treatment, the pandemic is associated with worsened mental health and difficulties in care access (61). Therefore, it is critical to investigate methods to improve treatment accessibility, particularly since the current study supports the effectiveness of these treatments in ameliorating clinical symptoms.

Lastly, 65% the study participants were female, and most participants were in their late 30’s. Generally, stigma within males is associated with negative attitudes towards treatments (62) and societal gender roles can also contribute to greater treatment-seeking in women (63). Although digital literacy has increased in the older population during the pandemic (64), navigating through online platforms may deter some less technologically-savvy individuals from using online psychotherapy. Therefore, it is critical for future studies to delineate methods to improve equitable access to care, including those delivered digitally (29, 65).

### 4.2. Clinical implications and future research

A real novelty of the study was its focus on shared decision-making in patient-centered care (65). The baseline difference observed in this study indicate that when given the choice, different participant profiles may be drawn to different forms of care. Specifically, patients with greater severity of depressive symptoms and lower quality of life were significantly more likely to opt for in-person therapy. Based on the baseline clinical scores, patients who selected in-person therapy were in the severe depression range, whereas those in the e-CBT group experienced moderate depression. Moreover, patients in the in-person CBT group had 17% lower quality of life scores at baseline than those in the e-CBT group. These patterns indicate that treatment preference may be influenced by MDD severity.

Since therapeutic alliance can predict treatment outcome (66), future studies should also consider methods to enhance the patient perception of the therapeutic relationship over the digital realm. Exploring this factor when developing online programs may be critical in patients with more severe MDD symptomatology, who may require higher intensity therapist support and stronger working alliance (67–69). Indeed, enhanced therapeutic support is associated with greater e-CBT completion rates (70). An RCT of e-CBT with and without therapist support observed a 30% difference in attrition rates for the therapist-supported group (20%) compared to the minimal support group (50%) (71). At the same time, online care delivery may lead patients to evaluate working alliance differently than in-person (72), even in the absence of such differences (73).

Given that nearly half of the participants chose online treatment and half chose in-person treatment, future studies may benefit from exploring participant perceptions and other factors like therapeutic rapport, that may contribute to individuals preferring one treatment delivery type over the other. A previous study noted the importance of addressing concerns regarding efficacy, privacy, data security, and motivation, in addition to therapist involvement, in online psychotherapy (74). The current findings support the importance of exploring how these changes affect treatment preferences and clinically meaningful treatment outcomes in different profiles within the target population (75). Strategic consideration of these factors may also improve the attrition rates observed in these gold-standard treatments and personalize treatments to cost-effectively meet the unique needs of individuals (76, 77).

## 5. Conclusion

Taken together, this study aimed to evaluate the efficacy of delivering a therapist-supported e-CBT program for MDD patients through a secure online platform. The findings demonstrated that in-person and e-CBT groups significantly improved depressive symptom severity and quality of life. E-CBT may be critical in addressing accessibility barriers and providing significant time savings to care, providers. However, MDD severity is a factor that should be considered when determining the most appropriate treatment delivery format for individuals seeking care.

## Data availability statement

The original contributions presented in the study are included in the article/Supplementary material, further inquiries can be directed to the corresponding author.

## Ethics statement

The studies involving human participants were reviewed and approved by Queen’s University Health Sciences and Affiliated Teaching Hospitals Research Ethics Board. Written informed consent for participation was not required for this study in accordance with the national legislation and the institutional requirements.

## Author contributions

NA and MO designed the study. EM, GG, FK, AmS, and MO conducted the data analysis. NA developed and implemented the CBT modules, which were edited by NA, JJ, and TG. AK, YS, SM, CSY, AnS, MG, ZA, MY, and AP administered CBT. EM drafted the first manuscript and subsequent drafts were edited by the entire research team. All authors contributed to the article and approved the submitted version.

## Conflict of interest

NA and MO cofounded the care delivery platform in use (i.e., OPTT) and have ownership stakes in OPTT Inc. AmS was employed by OPTT Inc.

The remaining authors declare that the research was conducted in the absence of any commercial or financial relationships that could be construed as a potential conflict of interest.

## Publisher’s note

All claims expressed in this article are solely those of the authors and do not necessarily represent those of their affiliated organizations, or those of the publisher, the editors and the reviewers. Any product that may be evaluated in this article, or claim that may be made by its manufacturer, is not guaranteed or endorsed by the publisher.

## Footnotes

^1^ clinicaltrials.gov/ct2/show/NCT04478058

^2^ www.optt.ca
