# Supplementary material for: Comparison of online and in-person cognitive behavioral therapy in individuals diagnosed with major depressive disorder: a non-randomized controlled trial
Source: Front Psychiatry. 2023 Apr 28;14:1113956. doi: 10.3389/fpsyt.2023.1113956 (PMC10175610; doi:10.3389/fpsyt.2023.1113956)
Supplement: Supplementary file 1 [file Data_Sheet_1.DOCX]

**COMPLETER ANALYSIS**

*Table 1. Mean, standard deviation, and ANOVA of primary outcomes as a function of 2 (CBT Delivery Type) by 3 (Time) design*

| **Variable** | **Condition** | **N** | **Baseline (0 Weeks)** | | **Mid-Treatment (6 Weeks)** | | **Post-treatment (12 Weeks)** | | **Outcome Analysis- ANOVA** | | | | | | | | |
| --- | --- | --- | --- | --- | --- | --- | --- | --- | --- | --- | --- | --- | --- | --- | --- | --- | --- |
|  |  |  | ***M*** | ***SD*** | ***M*** | ***SD*** | ***M*** | ***SD*** | ***Time*** | | | ***CBT Delivery Type*** | | | ***Time by CBT Delivery Type Interaction*** | | |
|  |  |  |  |  |  |  |  |  | df | F, p | ηp^2^ | df | F, p | ηp^2^ | df | F, p | ηp^2^ |
| PHQ-9 | IP-CBT | 24 | 21.25 | 5.80 | 18.17 | 6.23 | 15.71 | 5.20 | 2, 86 | 18.881, < 0.001 | 0.305 | 1, 43 | 12.686, 0.001 | 0.228 | 2, 86 | 1.337, 0.268 | 0.030 |
|  | e-CBT | 21 | 15.67 | 5.12 | 12.24 | 6.43 | 12.10 | 4.38 |  |  |  |  |  |  |  |  |  |
| QIDS-SR | IP-CBT | 25 | 19.96 | 4.75 | 18.48 | 4.20 | 16.40 | 3.81 | 2, 88 | 18.464, < 0.001 | 0.296 | 1, 44 | 22.774, < 0.001 | 0.341 | 2, 88 | 0.737, 0.482 | 0.016 |
|  | e-CBT | 21 | 13.81 | 4.78 | 13.29 | 4.15 | 11.38 | 4.24 |  |  |  |  |  |  |  |  |  |
| Q-LES-Q | IP-CBT | 24 | 31.75 | 11.63 | 34.29 | 12.53 | 36.88 | 13.24 | 1.701, 88 | 14.011, <0.001 | 0.242 | 1, 44 | 4.080, 0.049 | 0.085 | 1.611, 88 | 0.035, 0.947 | 0.001 |
|  | e-CBT | 22 | 37.55 | 7.77 | 39.73 | 7.86 | 42.82 | 6.19 |  |  |  |  |  |  |  |  |  |

*Note.* IP-CBT = in-person cognitive behavioural therapy; e-CBT = electronic cognitive behavioural therapy; M = mean; PHQ-9 = Patient Health Questionnaire; QIDS-SR = Quick Inventory of Depressive Symptomatology; Q-LES-Q = Quality of Life Enjoyment and Satisfaction; SD = standard deviation

*Table 2. Pairwise comparisons of Time for each outcome*

|  | **0 Weeks** | | **6 Weeks** | | **12 Weeks** | | **0 vs. 6 Weeks** | **6 vs. 12 Weeks** | **0 vs. 12 Weeks** |
| --- | --- | --- | --- | --- | --- | --- | --- | --- | --- |
|  | **x̄** | **SE** | **x̄** | **SE** | **x̄** | **SE** | **p, 95%CI[UL,LL]** | | |
| PHQ-9 | 18.46 | 0.82 | 15.02 | 0.94 | 13.90 | 0.72 | < 0.001, [1.43, 5.08] | 0.260, [-0.55, 3.15] | < 0.001, [2.53, 6.59] |
| QIDS-SR | 16.89 | 0.71 | 15.88 | 0.62 | 13.89 | 0.59 | 0.111, [-0.16, 2.16] | <0.001, [0.76,3.22] | <0.001, [1.65, 4.34] |
| Q-LES-Q | 34.65 | 1.47 | 37.01 | 1.56 | 39.85 | 1.55 | 0.005, [-4.12, -0.60] | 0.047, [-5.65, -0.26] | <0.001, [-7.84, 2.56] |

*Table 3. Pairwise comparisons of CBT Delivery Type for each outcome*

|  | **In-person CBT** | | **e-CBT** | | **p, 95%CI[UL,LL]** |
| --- | --- | --- | --- | --- | --- |
|  | ***x̄*** | ***SE*** | ***x̄*** | ***SE*** |  |
| PHQ-9 | 18.38 | 0.97 | 13.33 | 1.03 | < 0.001, [2.19,7.90] |
| QIDS-SR | 18.28 | 0.77 | 12.83 | 0.84 | < 0.001, [3.15, 7.76] |
| Q-LES-Q | 34.31 | 1.96 | 40.03 | 2.05 | 0.049, [-11.44, -0.013] |

**INTENTION TO TREAT ANALYSIS**

​

*Table 4. Mean, standard deviation, and ANOVA of primary outcomes as a function of 2 (CBT Delivery Type) by 3 (Time) design*

| **Variable** | **Condition** | **Baseline (0 Weeks)** | | | **Mid-Treatment (6 Weeks)** | | | **Post-treatment (12 Weeks)** | | | **Outcome Analysis- ANOVA** | | | | | |
| --- | --- | --- | --- | --- | --- | --- | --- | --- | --- | --- | --- | --- | --- | --- | --- | --- |
|  |  | ***N*** | ***M*** | ***SD*** | ***N*** | ***M*** | ***SD*** | ***N*** | ***M*** | ***SD*** | ***Time df (2, 212)*** | | ***CBT Delivery Type df (1, 106)*** | | ***Interaction df (2, 212)*** | |
|  |  |  |  |  |  |  |  |  |  |  | **F, p** | **ηp^2^** | **F, p** | **ηp^2^** | **F, p** | **ηp^2^** |
| **PHQ-9** | IP-CBT | 55 | 19.91 | 5.41 | 31 | 17.81 | 5.69 | 24 | 15.71 | 5.20 | 10.071, <0.001 | 0.088 | 25.97, <0.001 | 0.111 | 0.318, 0.728 | 0.003 |
|  | e-CBT | 52 | 16.08 | 5.09 | 30 | 12.97 | 6.21 | 23 | 12.48 | 4.49 |  |  |  |  |  |  |
| **QIDS-SR** | IP-CBT | 55 | 19.29 | 4.74 | 31 | 18.52 | 4.2 | 25 | 16.40 | 3.81 | 6.36, 0.002 | 0.001 | 54.103, <0.001 | 0.205 | 0.065, 0.937 | 0.001 |
|  | e-CBT | 51 | 14.61 | 4.78 | 32 | 13.41 | 4.28 | 22 | 11.86 | 4.71 |  |  |  |  |  |  |
| **Q-LES-Q** | IP-CBT | 55 | 32.09 | 9.83 | 32 | 35.66 | 12.00 | 24 | 36.88 | 13.24 | 5.62, 0.004 | 0.051 | 11.22, 0.001 | 0.052 | 0.218, 0.804 | 0.002 |
|  | e-CBT | 52 | 36.31 | 8.62 | 31 | 39.03 | 8.09 | 23 | 42.52 | 6.21 |  |  |  |  |  |  |

*Note.* IP-CBT = in-person cognitive behavioural therapy; e-CBT = electronic cognitive behavioural therapy; M = mean; PHQ-9 = Patient Health Questionnaire; QIDS-SR = Quick Inventory of Depressive Symptomatology; Q-LES-Q = Quality of Life Enjoyment and Satisfaction; SD = standard deviation

An unstructured mixed-effect ANOVA analysis was used to evaluate intent-to-treat analysis (with CBT-Type and evaluation times as fixed factors), which also included participants who did not complete the whole round of therapy. Similar to the completer analysis, a significant change in PHQ-9 score was observed in each group (p<0.001; Figure 1) and no significant difference between the effect size across the groups (p=0.497) was observed. Analysis also revealed a similar improvement in quality of life as evaluated by the Q-LES-Q (p<0.001; Figure 2), with no significant difference between the effect size across the groups (p=0.96). There were also significant improvements in QIDS-SR scores (p<0.001; Figure 3) and no significant difference between the effect size across the groups (p=0.67).

Significant main effects were observed for Time and CBT Delivery Type for all measures. An interaction between time and CBT delivery type could not be demonstrated for any of the outcome variables. At baseline, participants who opted for in-person therapy had significantly higher scores on the PHQ-9 (p < 0.001) and QIDS-SR (p < 0.001) and significantly lower scores on the Q-LES-Q (p = 0.027) than those in the e-CBT group. Participants in the in-person CBT group had significantly greater scores than those in the e-CBT group on the PHQ-9 (p < 0.001), QIDS-SR (p < 0.001), and significantly lower scores on the Q-LES-Q (p = 0.001). Irrespective of CBT delivery type, Bonferroni post-hoc analysis demonstrated significant differences in PHQ-9 scores at 0 weeks vs. 6 weeks (p = 0.008) and 0 weeks vs. 12 weeks (p < 0.001). QIDS-SR scores were significantly different at 0 weeks vs. 12 weeks (p = 0.001) but not at 6 weeks (p = 0.509). Lastly, Q-LES-Q scores were significantly different at 0 weeks vs. 12 weeks (p = 0.005) but not at 6 weeks (p = 0.135).

*Figure 1. Patient Health Questionnaire (PHQ-9) at three intervals of treatment, Weeks 0, 6 and 12, in both in-person and eCBT treatment conditions.*

*Figure 2. Quick Inventory of Depressive Symptomatology (QIDS-SR) at three intervals of treatment, Weeks 0, 6 and 12, in both in-person and eCBT treatment conditions.*

*Figure 3. Quality of Life Enjoyment and Satisfaction (Q-LES-Q) at three intervals of treatment, Weeks 0, 6 and 12, in both in-person and eCBT treatment conditions.*
